# Supplementary material for: Effects of Schistosoma haematobium infection and treatment on the systemic and mucosal immune phenotype, gene expression and microbiome: A systematic review
Source: PLoS Negl Trop Dis. 2024 Sep 9;18(9):e0012456. doi: 10.1371/journal.pntd.0012456 (PMC11412685; doi:10.1371/journal.pntd.0012456)
Supplement: S1 Table — (DOCX) [file pntd.0012456.s001.docx]

**S1 Table. List of excluded studies during quality assessment using the Downs and Black Checklist.**

| **#** | **First author, year, journal** | **Downs and Black Total Score (out of 28 possible points)** |
| --- | --- | --- |
| 2 | Eissa, 2005, Clinical Biochemistry | 14 |
| 1 | Ma, 2011, Biochemical and Biophysical Research Communications | 12 |
| 10 | Metwally, 2011, Cancer Cell International | 14 |
| 9 | Raziuddin, 1990, Cancer | 12 |
| 8 | Raziuddin, 1991, Journal of Clinical Immunology | 14 |
| 7 | Raziuddin, 1992, European Journal of Immunology | 12 |
| 6 | Raziuddin, 1992, Scandinavian Journal of Immunology | 13 |
| 3 | Thanan, 2012, Mediators of Inflammation | 13 |
| 5 | Ukwandu, 2001, Journal of Tropical Pediatrics | 13 |
| 4 | van Den Biggelaar, 2000, Journal of Infectious Diseases | 14 |
| 11 | Wu, 1998, Cancer Research | 12 |
