# Supplementary material for: Effects of Schistosoma haematobium infection and treatment on the systemic and mucosal immune phenotype, gene expression and microbiome: A systematic review
Source: PLoS Negl Trop Dis. 2024 Sep 9;18(9):e0012456. doi: 10.1371/journal.pntd.0012456 (PMC11412685; doi:10.1371/journal.pntd.0012456)
Supplement: S1 Text — (DOCX) [file pntd.0012456.s004.docx]

**S1 Text. Full search strategy.**

**ALL Complete Search Strategy**

The following is the comprehensive search strategy for the systematic review. It was first performed on December 22, 2022 and was re-run on August 9th, 2023.

PubMed (12/22/22)

(“Schistosoma haematobium"[Mesh] OR "Schistosomiasis haematobia"[Mesh] OR "Schistosoma haematobium") AND ("Immunity"[Mesh] OR "Immunomodulation"[Mesh] OR immun*[tiab] OR "Microbiota"[Mesh] OR microbio*[tiab] OR "Gene Expression"[Mesh] OR gene*[tiab] OR biomarker*[tiab] OR "Biomarkers"[Mesh] OR pathogen*[tiab])

Medline (12/22/22)

1. exp Schistosoma haematobium/

2. exp Schistosomiasis haematobia/

3. (schistosoma adj1 haematobium*).ti,ab.

4. ((urinary or urogenital) adj1 schistosomias*).ti,ab.

5. 1 or 2 or 3 or 4

6. exp Immunity/

7. exp Immunomodulation/

8. immun*.ti,ab.

9. exp Microbiota/

10. (microbio* or microflor*).ti,ab.

11. exp Gene Expression/

12. exp Biomarkers/

13. (gene* or biomarker* or (biological adj2 marker*) or pathogen*).ti,ab.

14. 6 or 7 or 8 or 9 or 10 or 11 or 12 or 13

15. 5 and 14

EMBASE (12/22/22)

1. exp Schistosoma haematobium/

2. exp schistosomiasis haematobia/

3. (schistosoma adj1 haematobium*).ti,ab.

4. ((urinary or urogenital) adj1 schistosomias*).ti,ab.

5. 1 or 2 or 3 or 4

6. exp immunity/

7. exp immunomodulation/

8. immun*.ti,ab.

9. exp microflora/

10. (microbio* or microflor*).ti,ab.

11. exp gene expression/

12. exp biological marker/

13. (gene* or biomarker* or (biological adj2 marker*) or pathogen*).ti,ab.

14. 6 or 7 or 8 or 9 or 10 or 11 or 12 or 13

15. 5 and 14

Scopus (12/22/22)

( TITLE-ABS-KEY ( immun* OR microbio* OR microflor* OR gene* OR biomarker* OR ( biological W/2 marker* ) OR pathogen* ) ) AND ( ( TITLE-ABS-KEY ( ( ( urinary OR urogenital ) W/1 schistosomias* ) ) ) OR ( TITLE-ABS-KEY ( ( schistosoma W/1 haematobium* ) ) ) )

Cochrane (12/22/22)

#1 MeSH descriptor: [Schistosoma haematobium] explode all trees

#2 MeSH descriptor: [Schistosomiasis haematobia] explode all trees

#3 (schistosoma NEAR/1 haematobium*)

#4 ((urinary or urogenital) NEAR/1 schistosomias*)

#5 #1 or #2 or #3 or #4

#6 MeSH descriptor: [Immunity] explode all trees

#7 MeSH descriptor: [Immunomodulation] explode all trees

#8 immun*

#9 MeSH descriptor: [Microbiota] explode all trees

#10 (microbio* or microflor*)

#11 MeSH descriptor: [Gene Expression] explode all trees

#12 MeSH descriptor: [Biomarkers] explode all trees

#13 (gene* or biomarker* or (biological NEAR/2 marker*) or pathogen*)

#14 #6 or #7 or #8 or #9 or #10 or #11 or #12 or #13

#15 #5 and #14
