# Supplementary material for: Effects of Schistosoma haematobium infection and treatment on the systemic and mucosal immune phenotype, gene expression and microbiome: A systematic review
Source: PLoS Negl Trop Dis. 2024 Sep 9;18(9):e0012456. doi: 10.1371/journal.pntd.0012456 (PMC11412685; doi:10.1371/journal.pntd.0012456)
Supplement: S2 Text — (DOCX) [file pntd.0012456.s005.docx]

**S2 Text. Decision rules for data selection and extraction processes.**

**Eligibility criteria for inclusion**

Studies of humans and experimental ex vivo or animals with *Schistosoma haematobium* infection and the impact on the immune system, gene expression or microbiome, with comparator groups that are likely to be humans or animals without schistosome infection, those that have been treated for schistosome infection, or by infection burden.

**Exclusion criteria**

Studies were excluded if they did not meet the above eligibility criteria or fell into at least one of the following categories:

1. Review pieces, opinion pieces, case studies, and case series
2. Outcomes not related to immune system, gene expression or microbiome
3. Diagnosis of schistosomiasis not confirmed by microscopic, antigen, molecular, or serologic
4. Not available in English
5. Presents a study protocol, not results
6. Unable to be obtained
7. Abstract without enough information and no full paper, such as conference/ meeting abstracts
8. Dissertations or theses
9. Non-peer reviewed online manuscripts
