# Supplementary material for: Effects of Schistosoma haematobium infection and treatment on the systemic and mucosal immune phenotype, gene expression and microbiome: A systematic review
Source: PLoS Negl Trop Dis. 2024 Sep 9;18(9):e0012456. doi: 10.1371/journal.pntd.0012456 (PMC11412685; doi:10.1371/journal.pntd.0012456)
Supplement: Attachment — Submitted filename: Response to Reviewers (point by point).docx [file pntd.0012456.s006.docx]

**Response to Reviewers**

**Manuscript: Effects of *Schistosoma haematobium* infection and treatment on the systemic and mucosal immune phenotype, gene expression and microbiome: A systematic review**

**Reviewer # 1’s Comments:**

Comment 1) The main objective of this manuscript is to systematically review the effects of S. haematobium infection on the immune system, gene expression, and microbiome of humans and experimental models. However, the current structure of the "results" section makes the audience easily lost due to numerous subsections and further subsections. Additionally, these sections are sometimes interconnected, making it challenging for readers to grasp the content. For example, sections starting from line 375, 703, 823, 942, 1043, 1132, 1206, and 1270 provide brief summaries of previous literature reviews. Presenting them as separate sections disrupts the reading flow. I suggest the authors either restructure the content or include a figure/table to illustrate the organization of results section.

Response to Comment #1 We thank the reviewer for this valuable comment and excellent suggestion. As suggested, we incorporated a table as an overview of how the results were organized in a newly generated table (table 1) in line 149 to which we refer to in line 148. Throughout the text, we have concluded each section when appropriate with a summary of the literature discussed to try to further improve the flow for the reader.

Comment 2) The authors should provide a more detailed explanation of the antigens studied in the literature they referenced. In Box 2, schistosome antigens include "soluble egg antigen," "adult worm antigen," "soluble adult worm antigen preparation," and "schistosome phosphatidylserine”. The authors frequently refer to "schistosome antigens" throughout the text (e.g., line 267, 269, etc.). Are they exactly the same molecules? Additionally, the authors also talked about egg-specific, cercaria-specific, and adult worm-specific antigens (line 320-331). What are the differences between stage-specific antigens and schistosome antigens? Clear and complete information about the antigens is crucial for readers to compare and evaluate the results.

Response to Comment #2: We express gratitude to the reviewer for providing such insightful feedback. We agree that it is imperative to be specific about the range of schistosome antigens, as each elicits a distinct immune response. Therefore, we have now meticulously specified the antigens employed in each study. To underscore the significance of delineating these various antigens, we have incorporated a paragraph into box 2 explaining their importance. Furthermore, we have made necessary revisions to the tables and text to enhance clarity and completeness where information was previously lacking or unclear (tables 2, 6, 7, 14; line 194, 202, 203, 209, 230, 232, 235, 250, 288, 289, 291, 292, 293, 311, 320, 345, 347, 349, 357, 358, 379, 384, 396, 400, 402, 514, 592, 594, 602, 672, 748, 771, 772, 783, 1249, 1281, 1284).

Comment 3) For the section "S. haematobium infection and host gene expression," it is unclear whether the observed changes were at the RNA or protein level. The authors should clarify this, for example, by using italics to indicate genes.

Response to Comment #3:

Thank you for this helpful comment. As suggested, we used italics to indicate genes and adjusted this in this in the results section (tables 11- 14; line 1049, 1050, 1051, 1053, 1054, 1056, 1059, 1060, 1063, 1070, 1071, 1074, 1076, 1105, 1138, 1142, 1159, 1160, 1162, 1168, 1201, 1206, 1211, 1212, 1242, 1244, 1245, 1253, 1254).

Comment 4) The authors should explicitly list in the tables and/or mention in the main text the studies that involve patients with not only schistosome infections but also multiple infections. This is crucial, as multiple infections can significantly confound our understanding of schistosome infections.

Response to Comment #4: We agree with this comment from the reviewer and added this pertinent information to the tables by adding an asterisk to the specific studies in the tables. Below the appropriate tables we identified the asterisk as “*Studies included participants with additional parasitic infections.”

Minor comments:

Minor comment 1) The current way of referencing studies in tables makes it difficult to locate the original study in the reference section. Please consider using the numerical indication that matches the reference style.

Response to minor comment #1: We thank the reviewer for this helpful suggestion. We implemented this by adding the appropriate reference numbers from the manuscript’s bibliography in the table under the first column “study”.

Minor comment 2) Line 205-207, not sure what “significantly different between the groups studied” refers to. Please revise.

Response to minor comment #2: Thank you for pointing out the need for clarification. We revised the sentence in line 219- 220 to clarify. We now state, “Factors like soil transmitted helminths (STH) (59) and filarial co-infections (60) were similarly distributed among the groups. Nevertheless, co-infections in these two studies might have influenced the results”.

Minor comment 3) Line 285, I suggest the authors use “purified protein derivate” rather than PDD. The term explanation is only in Box 2 and this term does not appear many times in the main text.

Response to minor comment #3: We agree with this comment and modified this appropriately in line 308.

Minor comment 4) Line 346-346, talking about HIV here feels out of place. Please consider revising.

Response to minor comment #4: We thank the reviewer for this comment. Since *S. haematobium* infection has been associated with HIV infection, we would prefer to keep this reference to HIV in place. We have added a sentence to explain why HIV is of importance in this context. We now write, “This is pertinent since some studies have shown associations between HIV and *S. haematobium* infections” in line 375- 376.

Minor comment 5) Line 439, what does "encounter" mean here? Does it refer to the first responsive immune cells or the first cell to come into contact with the parasite?

Response to minor comment #5: We clarified this in line 471- 473 that the first contact with the parasite is meant. We now write, “Since DCs are the first to encounter the parasite *Sh in all its forms, including eggs that migrate into tissue*, it has been hypothesized that they may play a part in the known T cell hyporesponsiveness occurring in chronic *Sh* infections (102)”.

Minor comment 6) Line 703, the section title should be revised to match the rest section title style. Same for line 1270.

Response to minor comment #6: Thank you for pointing this out. We have fixed the titles in line 757, 924-925 and 1220- 1221.

Minor comment 7) Line 754-747, this sentence is very long and hard to understand.

Response to minor comment #7: Thank you for this helpful comment. We modified the sentence accordingly (line 812- 814) and write, ”A recent study in Zambia by Sturt et al. evaluated cervical immune changes in women with definite FGS (positive vaginal or cervical *Sh* PCR), probable FGS (urinary *Sh* eggs or CAA with one of four typical cervical findings), and those without FGS (absence of these findings) (148)”.

Minor comment 8) Line 876-879, I suggest moving this sentence to the beginning of the section.

Response to minor comment #8: We agree with this comment and moved the sentence as suggested (line 939- 941).

Minor comment 9) Line 997, please use the full term of SCC as it first appears in the text.

Response to minor comment #9: Thank you for this suggestion. We have removed this abbreviation in the text for clarity (line 1069, 1073- 1074).

Minor comment 10) Line 987, “….Sh-associated..” to ““….Sh-associated..” Same for line 1076.

Response to minor comment #10: Thank you for this comment. We adjusted the manuscript accordingly throughout the manuscript.

Minor comment 11) Ensure consistent styling for "ex vivo" and "in vivo.  These words are italic in line 36, 661, and 948, but not in lien 868 and 1007.

Response to minor comment #11: We apologize for this error. We corrected the styling to italic for both "ex vivo" and "in vivo” throughout the manuscript.

Minor comment 12) Line 1146-1151, what are “the specific organ” and “most organs”?

Response to minor comment #12: Thank you for pointing out the need for calcification. We clarified which organs are referred to in line 1231- 1233. We now write, “Activity of GST and GR as well as GSH levels increased in the studied organs (liver, bladder, spleen and lung) at some timepoint post-infection followed by a partial decrease in the liver and bladder”.

**Reviewer # 2’s Comments:**

Comment 1) 1-Line 160: The subdivision of cytokine profiles of CD4+ T cells into simply Th1 and Th2 is quite anachronic for our present days (<https://doi.org/10.3389/fimmu.2023.1284178>), the T cell polarization landscape has greatly evolved and should be at least incorporated here. In fact, studies on Sh and the host immune responses do report on such updated T cell types (<https://doi.org/10.1093/infdis/jis524>; doi: 10.1093/infdis/jis654) and are counterintuitively reported in this review.

Response to Comment #1: Thank you for this valuable comment. We agree with the reviewer and modified the sentence which originally was in line 160, now line 166- 168. We now say, “CD4^+^ T helper (Th) cells produce a variety of cytokines that can be grouped based on the cells they are associated with, including Th1, Th2, Th17 and regulatory T cells cytokines”.

Comment 2) Several references mentioned in Box 1 do not appear adequate to support the points made eg. IL-2 can have pro- or anti-inflammatory properties depending on the immune milieu (38,39) where ref 38 = Lyke K, Dabo A, Sangare L, Arama C, Daou M, Diarra I, et al. Effects of concomitant Schistosoma haematobium infection on the serum cytokine levels elicited by acute Plasmodium falciparum malaria infection in Malian children. Infect Immun. 2006;74(10):5718–24; ref 39 = Ateba-Ngoa U, Adegnika A, Zinsou J, Kassa Kassa R, Smits H, Massinga-Loembe M, et al. Cytokine and chemokine profile of the innate and adaptive immune response of Schistosoma haematobium and Plasmodium falciparum single and co-infected school-1584 aged children from an endemic area of Lambaréné, Gabon. Malar J. 2015;14:94. Re-check throughout the manuscript and correct.

Response to Comment #2: Thank you for your thorough review and comment. We fixed all citations for box 2 and have now placed them below the box in line 173.

**Reviewer #3:**  
Comment 1) There are some instances of misspelling of Schistosoma e.g line 22

Response to Comment #1: We apologize for this oversight and have corrected the typo in line 22.

Comment 2) In the abstract 'Results' section, some more details on the findings would help the reader gain more insight.

Response to Comment #2: We appreciate the reviewer’s suggestion to enhance the abstract. We expanded the results section of the abstract as suggested. Specifically, we have elaborated on the genetic changes in the abstract in line 42- 44.

Comment 3) Line 73: Misspelling of 'predominately'

Response to Comment #3: We apologize for this and have corrected the typo in line 75 and 739.

Comment 4) To me it is surprising that only 2 studies were identified in PubMed, searching the same query on PubMed yields 1,320 results. Some clarification would be necessary here. There is also a typo in the supplementary materials, where the PubMed search string is missing at the start.

Response to Comment #4: Thank you for thoroughly assessing the supplemental materials of our manuscript. There was a leading quotation mark and parenthesis missing from the PubMed search. In addition, the PRISMA flow diagram was not correctly generated. This was now corrected in the supplementary materials and in figure 1 now displaying the 1304 results by PubMed search.

Comment 5) -Table 1 - misspelling of 'inflammatory' as 'inflamatory'

Response to Comment #5: We apologize for this mistake and corrected it accordingly in table 2 (formerly table 1).

Comment 6) It might be worth including a column for antigens used for stimulation to improve the readability of the table. This can also be more specific in the text e.g. line 369 and 1167, references to schistosome antigen should be more specific as to which antigen was used.

Response to Comment #6: We thank the reviewer for this thoughtful comment and feedback, which was also expressed by Reviewer 1. We have now added the specific schistosome antigen used throughout the manuscript, as suggested.

Comment 7) Similarly description of the sample types used for measurements would aid in the understanding of the studies, this is provided in some instances but is not comprehensive.

Response to Comment #7: Thank you for this comment and insightful suggestion. We incorporated the sample types used throughout the manuscript, where it was not mentioned before (line 211, 215, 225, 234, 243, 249, 252, 257, 258, 277, 289, 291, 297, 306, 321, 344- 345, 357, 371, 379, 392, 671, 1060, 1312, 1318- 1319)

Comment 8) Figure 2 legend - 'Sh-infected depending' - there is a word missing here

Response to Comment #8: Thank you for this helpful comment. We adjusted the sentence.

Comment 9) Line 823 - should be 'local tissue' not 'systemic'

Response to Comment #9: Thank you for your thoughtful review and noticing this mistake. We modified and corrected the title as suggested.

Comment 10) Line 877 - perhaps a comment should be added on mouse models using abdominal exposure to cercariae, do these not recapitulate natural Sh infection?

The included number of experimental animal studies is quite low, perhaps it would be beneficial to comment on the reasons for this

Response to comment #10: Thank you for this excellent point. Unfortunately, abdominal cercarial injection does not recapitulate natural *S. haematobium* infection. Abdominal cercarial injection does not lead to eggs migrating from the genitourinary plexus to the genitourinary/ reproductive organs as it does in humans. *S. haematobium* is specific to humans. We adjusted the manuscript to emphasize this (line 939- 943) and added reference 165. We now write, “Animal models that recapitulate natural *Sh* egg deposition during infection must be achieved by injection of eggs and/or cercariae into tissue, which is at least a partial limitation given that this technique disrupts the normal epithelium in a way that does not occur during natural human infection. To date no animal model exists that mimics the human cycle of *Sh* infection, which is likely due to its specificity to humans, unlike *Sm* which can infect rodents to provide a useful animal model (165)*”.*

Comment 11) Line 1126 - rephrase sentence e.g. 'were tested using whole blood ELISA, finding...'

Response to comment #11: We thank the reviewer for this suggestion and modified this in line 1303- 1305. We now write, “Individuals with *Sh* infection (n=15) who had lived in Spain for 6 months after immigrating from West Africa had increased *VEGF* expression on whole blood ELISA testing, which has been associated with bladder cancer, compared to those without *Sh* infection (n=18) (207)”.

Comment 12) Discussion Line 1297 - Here it would be good to comment on the lack of differences observed for CD8 T cells, this is an interesting point that is not highlighted sufficiently.

Response to comment #12: We thank the reviewer for this suggestion. Only two studies reported on CD8 T cells in blood. Nausch et al reported no difference in infected and uninfected while Nmorsi et al found increased CD8 T cell frequencies. We elaborate on this in the results section in the paragraph “CD4 and CD8 T cells”, specifically in line 601- 609. We chose to highlight this further in the discission section in line 1385-1386 with, “Interestingly, the role of CD8 in infection remains to be determined. One study showed no difference, while another indicated an increase in this pertinent T cell population systemically (103,123)”.

Comment 13) Line 1445 - should be antigen detection or testing etc.

Response to comment #13: We added ‘testing’ as the reviewer suggested (line 1534).

Comment 14) Though I would be cautious that the final figures (3 and 4) do not overstate the findings of one paper or another. I would suggest that the findings included in the summary figures be those that only have some kind of consensus from the reviewed papers (e.g. 2 or more), if this is not already the case.

Response to comment #14: We appreciate the feedback regarding these figures and appreciate the suggestion to be cautious. Given that a number of findings have only been studied once in this understudied area, we would like to include all findings in the figure. However, to address the reviewer’s excellent point, we have now modified figures 3 and 4 by coloring the findings light grey if the information is based on a single study.

Of note, we became aware of the importance of using person-first language in science as per the recommendations from the National Institute of Allergy and Infectious Diseases. This emphasizes individuals' humanity over their conditions, avoiding stigmatization and promoting inclusivity. For example, "person with schistosome infection" highlights that the individual is not solely defined by their medical condition. We have changed our language throughout the manuscript to reflect this.
