# Supplementary material for: Effects of Schistosoma haematobium infection and treatment on the systemic and mucosal immune phenotype, gene expression and microbiome: A systematic review
Source: PLoS Negl Trop Dis. 2024 Sep 9;18(9):e0012456. doi: 10.1371/journal.pntd.0012456 (PMC11412685; doi:10.1371/journal.pntd.0012456)
Supplement: Attachment — Submitted filename: Response to Reviewers (point by point).docx [file pntd.0012456.s007.docx]

**Response to Reviewers**

**Manuscript: Effects of *Schistosoma haematobium* infection and treatment on the systemic and mucosal immune phenotype, gene expression and microbiome: A systematic review**

**Reviewer Comments:**

The article has been revised to address the requested corrections; however, there are remaining ambiguities and flaws that require further resolution:

Comment 1: In Figure 1, a total of 6,973 studies are obtained from various databases/registers, while the sum of studies obtained from Embase, PubMed, MEDLINE, and Unspecified databases is 8,275?

Response to Comment #1:

We appreciate this comment and have revised the figure to show the total summing 6973. This was due to a switch in the way that Covidence was tracking sources that has now been reconciled as the “unspecified databases” have been removed.

Comment 2: In the case of Figures, the legend should be given below the Figures (Figure 1).

Response to Comment #2: We thank the reviewers for this pertinent observation. A legend was placed below figure 1.

Comment 3: In Figure 1, write the reason for excluding 293 articles in Figure 1. For example, 20 articles due to X; 50 articles due to Y (no need to write the verb).

Response to Comment #3: We appreciate the reviewer’s insightful comment and excellent suggestion. As recommended, we incorporated the reasons for exclusion in figure 1.

Comment 4: Scopus has been examined as a search database, however, it is not mentioned in Figure 1. Conversely, it is imperative to clearly identify unspecified databases in order to avoid any ambiguities.

Response to Comment #4: We thank the reviewer for this observation. We have now rectified this and the figure no longer includes unspecified databases. The revised PRISMA flow diagram now includes all relevant databases, including Scopus. We apologize for any ambiguity and greatly appreciate the careful review of our manuscript.

Comment 5: The "selection study" section should be divided into three separate sections: 1) Study selection 2) Quality assessment 3) Data extraction

Response to Comment #5: We appreciate the suggestion and have implemented the recommendation to divide the section into three distinct parts. However, since data extraction followed study selection during our process of study selection for this review, we have maintained the original sequence of these sections as follows: (1) Study Selection, (2) Data Extraction, and (3) Quality Assessment.

Comment 6: It is imperative to carefully read the entire manuscript for any potential writing issues. For example, in the "supplementary materials" section, the word "Pbmed" ought to be replaced with "PubMed."

Response to Comment #6: Thank you for this pertinent comment and the careful review. We updated and corrected the spelling of PubMed in the revised version of the supplemental materials and reviewed the entire document for errors.
