# Supplementary material for: Effects of Schistosoma haematobium infection and treatment on the systemic and mucosal immune phenotype, gene expression and microbiome: A systematic review
Source: PLoS Negl Trop Dis. 2024 Sep 9;18(9):e0012456. doi: 10.1371/journal.pntd.0012456 (PMC11412685; doi:10.1371/journal.pntd.0012456)
Supplement: Attachment — Submitted filename: Response to Reviewers (point by point).docx [file pntd.0012456.s008.docx]

**Response to Reviewers**

**Manuscript: Effects of *Schistosoma haematobium* infection and treatment on the systemic and mucosal immune phenotype, gene expression and microbiome: A systematic review**

**Reviewer Comments:**

The requested modifications by the authors have been made. The text appears to be well-written, however, unfortunately, there are still typographical errors in the text that need to be carefully read and corrected before any decision is made regarding this manuscript.
For examples:
Line 22, comma (,) should be removed.
Line 39, comma (,) should be removed.
Line 40, ....with (a) predominant type 2 ....is correct.
Line 57, ........infections(,) which (cause) urogenital.........is correct.
Line 1495, ......using Graph(P)ad Prism (software)....is correct.
The text before and after line 1495 has not been examined, and it is necessary for the authors to carefully do it.

We apologize for the typographical errors and thank the reviewers for these careful observations and corrections. We corrected the above as suggested. After close review of the manuscript before and after line 1495 the following additional modifications have been made:

- Line 73, comma added
- Line 77, comma added
- Line 103, comma added
- Line 123, comma added
- Line 129, comma added
- Line 134, comma added
- Line 141 comma added
- Line 143, comma added OR removed
- Line 145, corrected “*Sh* endemic” to “*Sh*-endemic”
- Line 146, comma added
- Table 1, comma added
- Line 151, comma added
- Line 154, comma added
- Line 155, comma added
- Line 156, comma added
- Line 165, comma added
- Line 166, comma added
- Line 174, comma added
- Line 186, corrected “2” to “two”
- Line 190, comma removed
- Line 196, comma removed
- Line 197, comma removed
- Line 204, comma removed
- Line 207, comma removed
- Line 211, corrected “Th2 related” to “Th2-related”
- Line 212, comma added
- Line 213, corrected “soil transmitted” to “soil-transmitted”
- Line 217, comma added
- Line 226, comma added
- Line 236, corrected “but no” to “without”
- Line 239, corrected “immunomodulatory” to “immune-modulatory”
- Line 256, deleted space between “6- 60”
- Line 273 added “to”
- Line 287, corrected spelling of “derivative”
- Line 312, comma added
- Line 314, corrected “pro- and anti-inflammatory” to “pro- or anti-inflammatory”
- Line 324, comma added
- Line 366, comma added
- Line 381, corrected “two year” to “two-year”
- Line 468, added “of”
- Line 516, corrected “two year” to “two-year”
- Line 780, comma added
- Line 782, comma added
- Line 795, comma added
- Line 807, comma removed
- Line 818, comma added
- Line 820, comma added
- Line 827, two commas added
- Line 795, comma added
- Line 795, comma added
- Line 795, comma added
- Line 821, corrected “of to “in”
- Line 865, comma added
- Line 868, comma added
- Line 871, comma added
- Line 879, comma added
- Line 884, comma added
- Line 887, comma added
- Line 891, deleted “(8)”
- Line 896, comma added
- Line 898, comma added
- Line 901, comma added
- Line 903, comma added
- Line 906, added “type”
- Line 909, comma added
- Line 917, comma added
- Line 920, comma added
- Line 929, comma added
- Line 931, comma added
- Line 1024, corrected “n” to “in”
- Line 1029, corrected “and” to “or”
- Line 1035, corrected “&” to “and”
- Line 1043, corrected “of” to “in”
- Line 1045, corrected “as” to “to be”
- Line 1047, corrected “of” to “in”
- Line 1047, corrected “mediator which is present only at low” to “mediator that is present only at low”
- Line 1063, comma added
- Line 1065, comma added
- Line 1065, corrected “of” to “in”
- Line 1069, comma added
- Line 1078, comma added
- Line 1084, comma added
- Line 1086, comma added
- Line 1088, comma added
- Line 1115, comma added
- Line 1123, comma added
- Line 1135, comma added
- Line 1139, comma added
- Line 1147, comma added
- Line 1168, comma added
- Line 1177-1178, corrected “as well as time the point” to “as well as the time point”
- Line 1179, comma added
- Line 1180, space added
- Line 1182, comma added
- Line 1184, comma added
- Line 1184-1195, corrected “upregulation of proliferation of lymphocytes” to “upregulation of lymphocyte proliferation”
- Line 1200, comma added
- Line 1202, added “that”
- Line 1218, added “of”
- Line 1221, comma added
- Line 1225, comma added
- Line 1228, comma added
- Line 1228, added “a”
- Line 1233, comma added
- Line 1233, corrected “increase” to “increases”
- Line 1233, deleted “increase”
- Line 1238, added “the”
- Line 1239, deleted “to”
- Line 1240, comma added
- Line 1243, comma added
- Line 1243, added “the”
- Line 1273, comma added
- Line 1279, corrected “has” to “have”
- Line 1280, added “in”
- Line 1284, comma added
- Line 1284, comma added
- Line 1286, comma added
- Line 1317, comma added
- Line 1332, comma added
- Line 1339, corrected “increase risk of the acquisition and transmission” to “increase the risk of acquisition and transmission”
- Line 1346, corrected “anti viral” to “anti-viral”
- Line 1367, comma added
- Line 1370, corrected “More” to “Moreover”
- Line 1373, comma added
- Line 1381, comma added
- Line 1383, corrected “for” to “to”
- Line 1386, added “the”
- Line 1393, comma added
- Line 1397, comma added
- Line 1399, comma added
- Line 1400, corrected “of” to “in”
- Line 1417, comma added
- Line 1419, comma added
- Line 1421, comma added
- Line 1428, corrected “affects to “affect”
- Line 1430, comma added
- Line 1436, comma added
