# Supplementary material for: Effects of Schistosoma haematobium infection and treatment on the systemic and mucosal immune phenotype, gene expression and microbiome: A systematic review
Source: PLoS Negl Trop Dis. 2024 Sep 9;18(9):e0012456. doi: 10.1371/journal.pntd.0012456 (PMC11412685; doi:10.1371/journal.pntd.0012456)
Supplement: Attachment — Submitted filename: Response to Reviewers (point by point).docx [file pntd.0012456.s009.docx]

**Response to Reviewers**

**Manuscript: Effects of *Schistosoma haematobium* infection and treatment on the systemic and mucosal immune phenotype, gene expression and microbiome: A systematic review**

**Reviewer Comments:**

The scientific aspects of this manuscript have been properly revised, however, there are still grammatical and writing issues that need to be addressed before a decision is made regarding this manuscript (i.e. Figure 2: the title of the figures should be written below it (i.e. legend). Additionally, incorrect words such as "Graphpad" should be avoided in Figure 2. etc). The entire manuscript must be thoroughly examined and not limited to the mentioned points only.

We apologize for any errors and thank the reviewers and editor for their careful observations and corrections. After a thorough review, we have made the following modifications to the manuscript:

- All titles of figures were moved below the figures as suggested.
- In figure 2 “Graphpad” was corrected to “GraphPad”.
- In figure 2 extra spacing was removed.
- In figure 3 “Biorender” was corrected to “BioRender”.
- In figure 4 “Biorender” was corrected to “BioRender”.
- Redundant spacing was removed in tables 2, 3, 4, 6 and 7.
- In table 14 space was added in the line describing the study by Osakunor et al.
- In table 10 in the row describing Hsieh’s study, “UTi” was corrected to “UTI”.
- In box 1 “and” was corrected to ”or”.
- In box 1 “IL-6 plays is central to host defense and is a potent induced of inflammation and acute phase proteins.” To “IL-6 is central to host defense and is a potent induced of inflammation and acute phase proteins.”
- In line 1025, “*c-KITi*” was corrected to “*c-KIT*”
- In line 1067, a comma was added after “*Sh* infection”.
- In line 1399 we changed the wording from “What is clearly evident from this review” to “What is clear from this review”.
- In the methodology section in line 1496 “Biorender” was corrected to “BioRender”.
